# Synthesis of multifunctional activated carbon nanocomposite comprising biocompatible flake nano hydroxyapatite and natural turmeric extract for the removal of bacteria and lead ions from aqueous solution

**DOI:** 10.1186/s13065-018-0384-7

**Published:** 2018-02-21

**Authors:** H. D. A. Chathumal Jayaweera, Induni Siriwardane, K. M. Nalin de Silva, Rohini M. de Silva

**Affiliations:** 1 Department of Chemistry, University of Colombo, Colombo, 00300 Sri Lanka; 2 Sri Lanka Institute of Nanotechnology (SLINTEC), Nanotechnology and Science Park, Mahenwatta, Pitipana, Homagama, 10200 Sri Lanka

**Keywords:** Hydroxyapatite, Turmeric extract, Activated carbon, Nano composites, Adsorption

## Abstract

Clean water, which is free from pathogens and toxic chemicals, is vital to human health. The blue planet is encountering remarkable challenges in meeting the ever-increasing demands of clean water. The intention of this research study was to develop a water filter material that is capable of removing bacterial contaminants and heavy metals from fresh water using cost effective and easily fabricated biocompatible filter material. For this purpose, granular activated carbon (GAC) was coated with both hydroxyapatite (HAP) nanoflakes and turmeric extract (TE) (HAP/TE/GAC) which had been extracted from natural turmeric powder. In addition, GAC was coated only with HAP nanoflakes to synthesize HAP coated GAC (HAP/GAC) composite. Prepared HAP/GAC and HAP/TE/GAC were characterized using Fourier-transform infrared spectroscopy, X-ray diffractometry, scanning electron microscopy and UV–visible spectrophotometry. Antibacterial effect of the prepared nanocomposites, HAP/GAC and HAP/TE/GAC was compared with neat GAC using Gram-negative bacteria *Escherichia coli*. Results showed that antibacterial studies of the synthesized nanocomposites exhibit effective antibacterial activity against *E. coli* compared with neat GAC alone. However, the composite HAP/TE/GAC revealed better activity than HAP/GAC. Heavy metal adsorption ability of the synthesized composites was carried out using Pb^2+^ ions at room temperature at different time intervals and different pH levels. The equilibrium adsorption data were assessed via Langmuir and Freundlich adsorption isotherm models for neat GAC, HAP/GAC and HAP/TE/GAC at pH 6. The equilibrium adsorption data for GAC, HAP/GAC and HAP/TE/GAC were well fitted with both Freundlich and Langmuir isotherm models in the given Pb^2+^ concentrations. The HAP/TE/GAC composite is capable of maintaining the natural function of GAC in addition to removal of bacterial contaminants and heavy metals, which can be used as a point-of-use water filter material.

**Electronic supplementary material:**

The online version of this article (10.1186/s13065-018-0384-7) contains supplementary material, which is available to authorized users.

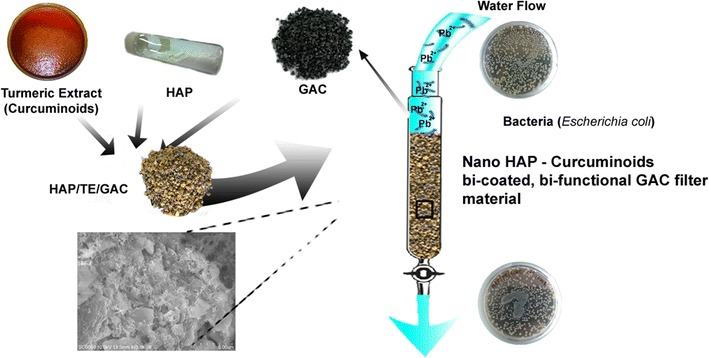

## Introduction

Water is one of the vital enablers of life on earth. Water envisioned for human consumption is universally accepted to be free from chemical constituents and micro-organisms in amounts which would provide a hazard to health. For a healthy person around 4–6 L of drinking water is the recommended amount and therefore it is extremely important to get access to clean water free from both chemical and biological contaminants. Consequently, purification of water for drinking purpose has become an important area of attention in the world [1]. Through purification removal of turbidity, organic contaminants, microorganisms and heavy metals from potable water sources are expected. In the recent past, there is an increased use of metallic substances in industries and this has resulted an accumulation of heavy metals in aquatic environment contaminating both surface and underground water sources [2]. It has been frequently observed that the amount of heavy metals such as lead (Pb), arsenic (As), cadmium (Cd), mercury (Hg), zinc (Zn), chromium (Cr), copper (Cu), silver (Ag) and iron (Fe) get accumulated due to most of the industrial actions [3, 4]. These heavy metals are known as ‘silent killers’ as this leads to many health problems including hyperpigmentation, hypopigmentation, kidney and gastrointestinal tract related disorders [3]. In addition, heavy metals are known to cause cancerous effect and has been observed in many occasions [5]. Of these heavy metals, Pb is one of the most abundant pollutant and this has been the subject of many research. Lead is known to cause many diseases leading to impaired fertility, mortality due to cardiovascular diseases and variety of neurodevelopmental outcomes [3, 6]. According to the World Health Organization (WHO) the provisional guideline value for lead is 0.01 mg/L [3]. On the other hand, it is widely observed that the presence of biological contaminants such as viruses, bacteria and protozoans in contaminated water lead to severe health effects in human. Often found types of viruses include Hepatitis A & E [3], Enterovirus [3] and Adenovirus [3, 7]. As it for bacteria *Escherichia coli* [3, 7], *Vibrio cholera* [3], *Salmonella* species [7] and *Shigella* [3, 7] are among the most common types available in contaminated water. In addition, protozoans like *Cryptosporidium* species [7] and *Giardia* species [7] can also be found in drinking water. Generally, *E. coli* is being considered as the indicator bacteria for the water quality [3]. Several chemical and physical agents are being used to remove microorganisms from drinking water. Most of the disinfectants are strong oxidants and therefore during their course of oxidation there is a tendency to occur harmful byproducts [7] which could damage healthy cells. As a result, the use of these disinfectants are limited and the attention is shifted to those that are not strong oxidants. As far as removal of heavy metals are concerned, several techniques such as precipitation [8, 9], reduction, ion-exchange [10, 11], adsorption [12, 13], electrochemical processes [14, 15] and reverse osmosis [16, 17] have been used. In recent past, the consideration has shifted towards nanomaterials in water purification due to their super sorption ability. On this regard, many nanomaterials, namely, iron oxide [18, 19], ZnO [20, 21], hydroxyapatite (HAP) [22], carbon nanotubes (CNT) [23–25] are used to remove heavy metals. Of which HAP records a greater potential in removing several heavy metals from contaminated water as well as exhibits a considerable amount of antibacterial property [22, 26]. In addition, many other engineered nanomaterials such as, Ag [27], TiO_2_ [28] and C_60_ [29, 30] derivatives have demonstrated strong antimicrobial properties. The application of these materials are limited mainly due to toxicity caused due to the production of reactive oxygen species and various other effects [29].

Even though, there are many methods available to eliminate heavy metals and biological contaminants separately, the methods available to eliminate both of these contaminants simultaneously are scarce [26, 31].

Activated carbon has attracted as one of the best adsorbent materials due to its capacity to remove unpleasant tastes, odors, color and various chemicals from water [32]. The mechanism of adsorption in activated carbon differs for organic and inorganic contaminants [33]. Although activated carbon efficiently removes majority of organic contaminants, it is less effective in removing many heavy metals and micro-organisms in water [34]. In our previous study, the possibility of in situ coating of HAP nanorods on activated carbon and Pb^2+^ adsorption property was demonstrated [22]. Studies on bi-coated HAP and curcumin disclosed the removal of both heavy metals and microorganisms such as *E. coli* and *Staphylococcus aureus* from contaminated water [26, 31]. Turmeric powder, from the rhizome of the plant *Curcuma longa*, is a well-known ingredient in many oriental cuisines and contains hundreds of molecular constituents with diverse biological activities. Out of which, twelve molecules show anti-inflammatory whereas twenty molecules reveal antibiotic activity, while ten molecules are known to be anti-oxidants. In addition, there are 14 molecules displaying cancer preventive properties [35, 36]. The components of turmeric are called curcuminoids and usually 3–5% of curcuminoids are there in its raw state [35, 37]. Dicinnamoylmethane derivatives are the three main golden color components present in different proportions in curcuminoids. These includes curcumin, demethoxycurcumin and bisdemethoxycurcumin [37]. Generally, curcuminoids are water insoluble in acidic and neutral pH whereas soluble in alkali solutions. However, soluble in oils such as castor oil, peanut oil and ethyl oleate [38]. Out of the three main components, antibacterial effect of curcumin is known to exert via inhibiting the bacterial endotoxin induced cytokines secretion and related activation mechanisms, this leads to direct suppressing of bacterial cell growth [39]. The objective of our work is to develop a low cost biocompatible filter material consisting of both metal adsorption and antibacterial properties. In order to accomplish this HAP coated granular activated carbon (HAP/GAC) is further treated with turmeric extract. This turmeric covered HAP coated granular activated carbon (HAP/TE/GAC) was tested on both Pb^2+^ and *E. coli* bacteria.

## Materials and methods

Calcium nitrate tetrahydrate—Ca(NO_3_)_2_·4H_2_O [Techno Pharmchem, Bahadurgarh, Haryana, India (30607)] was used as the calcium source, di-sodium hydrogen orthophosphate anhydrous LR—Na_2_HPO_4_ [S. D. Fine-Chem LTD (40158)] was used as the phosphate source with NH_3_ solution—(MW = 17.03 g/mol, density = 0.91 g cm^−3^, purity = 25% W/W, Merck Limited, Mumbai). Turmeric powder and GAC (Jacobi aquasorb AC) was used for the synthesis of the filter material. Distilled acetone was used as the solvent to prepare the extract from turmeric powder. When the solvent was not specified, double distilled water was used as the solvent. In order to prepare the Pb^2+^ solution, Pb(NO_3_)_2_ from Sigma-Aldrich was used and for the adjustment of pH hydrochloric acid from Daejung was used.

The solid reactants and the prepared nanocomposites were weighed using the analytical balance (CAS Cay 120). Drying was done using the electrical oven (Heraeus ST 6120). For the sonication purposes, sonicator (Sonorex super RK 1028 CH, BANDELIN Electronics, Berlin) was used. Fourier-transform infrared (FT-IR) spectra were obtained from Varian 660-IR, USA and KBr pellet technique was used to obtain the FT-IR spectra over the range of 400–4000 cm^−1^. The preparation of the pellet was done by mixing 2 mg of the sample with 200 mg of oven dried spectroscopy grade KBr (Sigma-Aldrich). The UV–visible spectra were obtained from GENESYS 10S UV–Vis and the samples for the UV–visible spectroscopy were prepared by dissolving small portion of the precipitate in distilled ethanol. The surface morphology and the microstructural characteristics of the synthesized nanocomposites were analyzed using scanning electron microscope (HITACHI SU6600). X-ray diffraction analysis of the synthesized HAP/GAC and HAP/TE/GAC nanocomposites were performed on a Bruker D8 Focus X-ray powder Diffractometer using CuKα radiation (λ = 0.154 nm) over a 2ϴ range of 3°–70°, with a step size of 0.020° and a step time of 1 s. The atomic absorption spectroscopy experiments were carried out using the instrument GBC 932 Plus flame atomic spectrometer.

The microbiology experiments were performed inside the Laminar Hood (Yamato, model ADS 160). The media for the bacterial growth were autoclaved using an Autoclave machine (KT-30SD, ALP Co. Ltd.) prior to the use. Glassware including petri plates were incubated at 180 °C for 2 h in an oven (Memmert Beschickung loading model 100–800) before use. Bacteria inoculated plates were incubated at 37 °C in an incubator (Memmert-Beschickung, model 100–800) for 18 h for growth.

### Synthesis of HAP coated granular activated carbon

The coating of HAP on granular activated carbon was carried out using an in situ method. GAC (12.0 g) was added into a 20 mmol of Ca(NO_3_)_2_•4H_2_O (0.4 M) solution. The temperature of the mixture was maintained at 80 °C. The mixture was stirred and a solution of 12 mmol Na_2_HPO_4_ (0.16 M) was added in a drop wise manner into the mixture. The pH of the mixture was maintained at 10 with the drop wise addition of conc. NH_3_. Mixture was then vigorously stirred at its boiling point for about 3 h. The reaction mixture was then aged for about 24 h in room temperature. The product obtained was suction filtered and washed thoroughly with double distilled water until the pH of the filtrate become neutral. The washings were tested for Ca^2+^ ions in HAP using atomic absorption spectroscopy. The prepared HAP coated granular activated carbon was kept in the oven at 80 °C for about 3 h. The synthesized nanocomposite was characterized using FT-IR spectroscopy, scanning electron microscopy and X-ray diffractometry (XRD) analysis.

### Preparation of turmeric extract from turmeric powder

Acetone was used as the solvent for the preparation of turmeric extract from turmeric powder. Distilled acetone (100 mL) was added into a portion of turmeric powder (60.0 g) and the mixture was sonicated at 50 °C for about 30 min. Then the mixture was suction filtered and the filtrate was collected. The product obtained after evaporating the solvent was an orange brown color residue. The turmeric extract was then characterized using FT-IR spectroscopy and UV–visible spectroscopy.

### Synthesis of turmeric extract coated, HAP impregnated granular activated carbon

The nanocomposite synthesized was further coated with the turmeric extract. For this turmeric extract (3.0 g) was dissolved in acetone (50 mL) and added in a drop wise manner to the mixture immediately after the complete addition of 12 mmol Na_2_HPO_4_ (0.16 M) solution in the preparation of HAP coated GAC composite as given above. The pH of the mixture was maintained around 7. The washings were tested using UV–visible spectroscopy for the leaching of TE. The bi-coated nanocomposite obtained was characterized using FT-IR spectroscopy and scanning electron microscopy.

### Filtering of an *Escherichia coli* bacterial suspension using column technique

The *E. coli* bacterial stock suspension was prepared by dissolving, isolated *E. coli* colonies from the previously prepared streaked plates, in 50 mL of double distilled autoclaved water. A sterile loop was used for transferring of bacteria. From the *E. coli* bacterial stock suspension, 100 times diluted bacterial suspension was prepared for the filtration. Lysogeny Broth (LB) Agar was used for the preparation of the culture media. The culture media was prepared by dissolving 25 g of LB and 20 g of Agar in 1 L of double distilled water.

Prior to filtering the *E. coli* bacterial suspension, GAC and the synthesized nanocomposites were autoclaved. Then 5 g of GAC, HAP/GAC and HAP/TE/GAC nanocomposites were packed into three separate columns with 12 mm diameter. The filter material was wetted with double distilled autoclaved water prior to filtering the *E. coli* bacterial suspension. A portion of 100 mL of the bacterial suspension was allowed to drain through the filter in the rate of 0.5 mL/min. The filtrate was collected in 10 mL fractions into autoclaved test tubes and kept in an ice bath to inhibit the bacterial growth. The *E. coli* bacterial content of these water samples was analyzed using the spread plate method, where 100 µL of each of the filtered fraction was spread in 15 mL of previously prepared LB Agar plate.

### Heavy metal adsorption

Adsorption studies were carried out for Pb^2+^ ions in order to figure out the optimum adsorption conditions and adsorption capacity using adsorption isotherms. The Pb^2+^ standard stock solution (1000 ppm) was prepared using lead nitrate Pb(NO_3_)_2_. The other Pb^2+^ solutions, which were used for the studies, were prepared with necessary dilutions using Pb^2+^ standard stock solution. The adjustment of pH was done using dropwise addition of hydrochloric acid.

The effect of pH on the adsorption capabilities of neat GAC and the synthesized GAC nanocomposites was studied in the pH range of 4–7. The concentration of the Pb^2+^ ion solution (1000 ppm), amount of the adsorbent (1.0 g) and the volume (100.0 cm^3^) were kept constant for 180 min of contact time with random stirring. The experiment was performed under room temperature.

The effect of contact time on the adsorption capacities of GAC and the synthesized nanocomposites was investigated by using a known concentration of Pb^2+^ ion solution (1000 ppm, 100.0 cm^3^) in the time span of 15–180 min. The temperature was kept constant at room temperature with random stirring. The adsorbent was subjected to filtration from the solution and the filtrate was analyzed using atomic absorption spectroscopy for the residual Pb^2+^ ion concentration. The adsorption capacity was evaluated using the general equation given below.

1 $$Q_{e} \; = \;\frac{{(C_{o} - C_{e} )V}}{m}$$

where *Q*_*e*_ is the amount of Pb^2+^ adsorbed on the adsorbent (mg/g) *C*_*o*_ and *C*_*e*_ are the Pb^2+^ concentration in solution before and after the adsorption (mg/dm^3^) respectively, *m* is the amount of matter in the reaction mixture (g) and *V* is the volume of the medium (dm^3^).

Adsorption isotherms analysis of Pb^2+^ ions with GAC and the synthesized nanocomposites were carried out by keeping a constant amount of adsorbent (1.0 g) and volume (100.0 cm^3^) of different initial concentration of Pb^2+^ ion solution. The Pb^2+^ ion solutions with different initial concentrations (400, 500, 600, 700 ppm) were prepared by using the 1000 ppm standard stock solution. The pH of the solutions was maintained at pH 6 (experimentally determined optimum pH for the studies) and the solution mixture was equilibrated for 165 min (experimentally determined contact time for the analysis). The adsorbent was filtered and the amount of Pb^2+^ ions in the filtrate was determined by AAS after necessary dilutions.

The adsorption equilibrium data for Pb^2+^ ions on GAC and the synthesized nanocomposites were analyzed using the Freundlich and Langmuir isotherm models. The Freundlich isotherm is given by Eq. (2) where *Q*_*e*_ is the amount adsorbed per unit weight of the adsorbent (mg/g), *C*_*e*_ is the equilibrium metal ion concentration in the solution (mg/dm^3^) and *K*_*f*_ and *n* are Freundlich isotherm constants, related to the adsorption capacity and adsorption intensity respectively [40, 41].

2 $$\log Q_{e} \; = \;\log K_{f} \; + \;\frac{1}{n}\log C_{e}$$

The graph of log *Q*_*e*_ vs log *C*_*e*_ plotted to determine the Freundlich isotherm constants.

The Langmuir isotherm linearized form written as given in Eq. (3) where the constants *q*_*m*_ and *K*, are related to the adsorption capacity and the energy of adsorption, respectively. The graph of *C*_*e*_/*Q*_*e*_ vs *C*_*e*_ was plotted to determine the Langmuir isotherm constants [42, 43].

3 $$\frac{{C_{e} }}{{Q_{e} }}\; = \;\frac{1}{{q_{m} K}}\; + \;\frac{{C_{e} }}{{q_{m} }}$$

## Results and discussion

### Characterization of HAP and HAP coated GAC

**Fig. 1:**
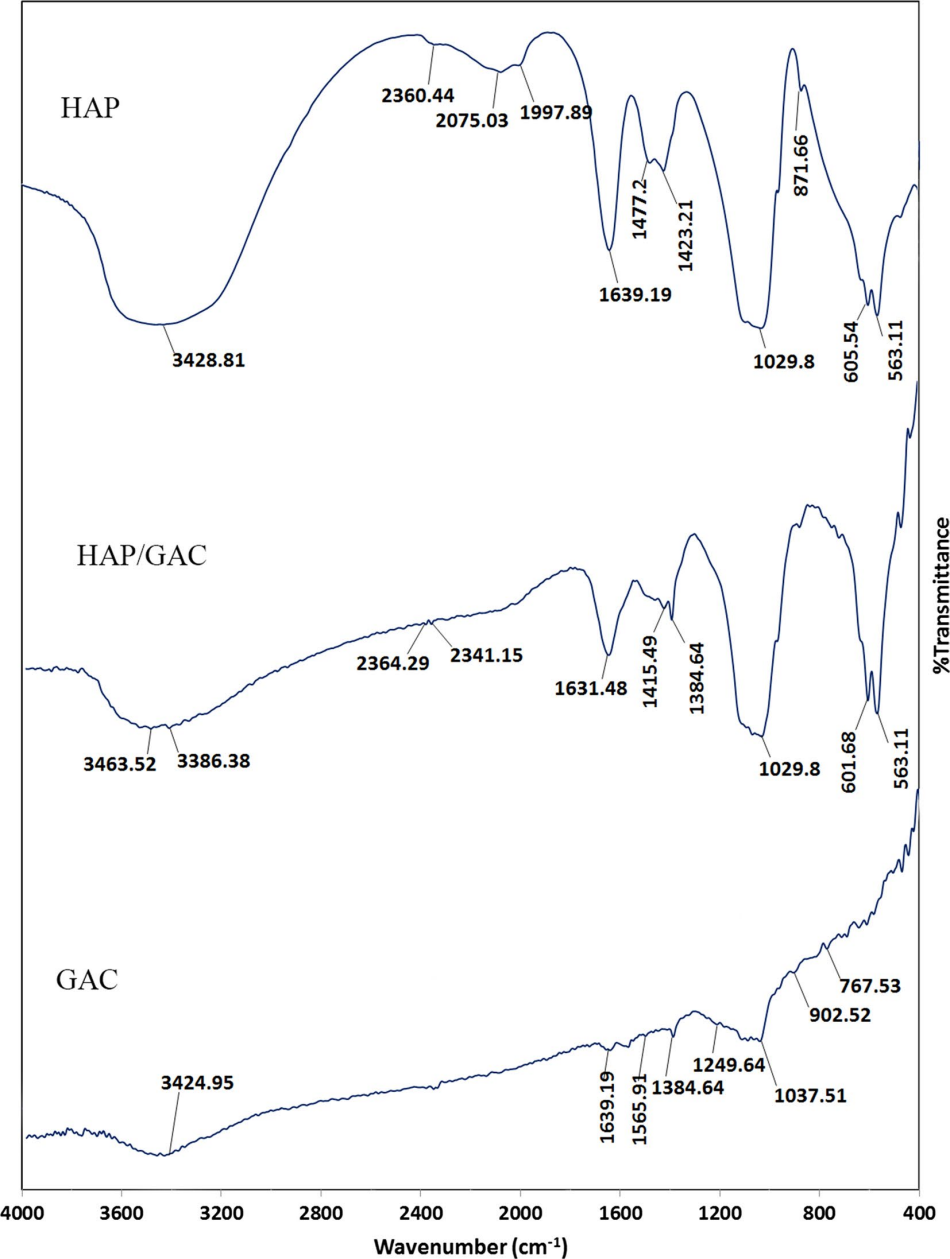
Comparison of FT-IR spectra of HAP, GAC and HAP/GAC

The formation of HAP coated GAC was confirmed by recording the FT-IR spectra and analyzing the characteristic peaks corresponding to the functional groups present in both HAP and GAC. Figure 1 shows the comparison of FT-IR spectra obtained for GAC, HAP and HAP coated GAC.

In the FT-IR spectrum of GAC (Fig. 1-GAC), the broad peak that appears at 3200–3600 cm^−1^ region accounts for O–H stretching vibration in hydroxyl groups. The band at 1639 cm^−1^ is ascribed to C=O stretching vibration in carboxylic groups. The peak in the region of 1565 cm^−1^ is attributed to the C=C stretching mode in aromatic rings [44]. The sharp peak observed at 1384 cm^−1^ accounts for C–O–H stretching in carboxylic groups. The peaks between 1249 and 1037 cm^−1^ are assigned to C–O stretching vibration in alcohol and phenol groups. The weaker bands between 902 and 767 cm^−1^ are ascribed to C–H bending in the aromatic rings [44].

The FT-IR spectrum of HAP (Fig. 1-HAP), the broad peak observed at 3700–3200 cm^−1^ is attributed to the stretching vibration mode of hydrogen bonded O–H and the peak at 1639 cm^−1^ is due to adsorbed H_2_O molecules. The peaks at 563, 960, 1029, 1083, 2360 cm^−1^ are assigned to PO_4_^3−^ group stretching mode and the peaks at 605, 871 cm^−1^ accounts for the bending mode of PO_4_^3−^ group [45, 46]. Thus, from analyzing the characteristic IR peaks in the FT-IR spectrum of HAP, it can be stated that the synthesized nanoflakes were HAP. The FT-IR spectrum of HAP coated GAC (Fig. 1-HAP/GAC) shows all prominent peaks at 563, 601, 1029 cm^−1^ corresponding to HAP nanoflakes. Thus, it can be stated that GAC has been coated with HAP to give HAP/GAC nanocomposites. In addition, the composite was further characterized using SEM and XRD.

**Fig. 2:**
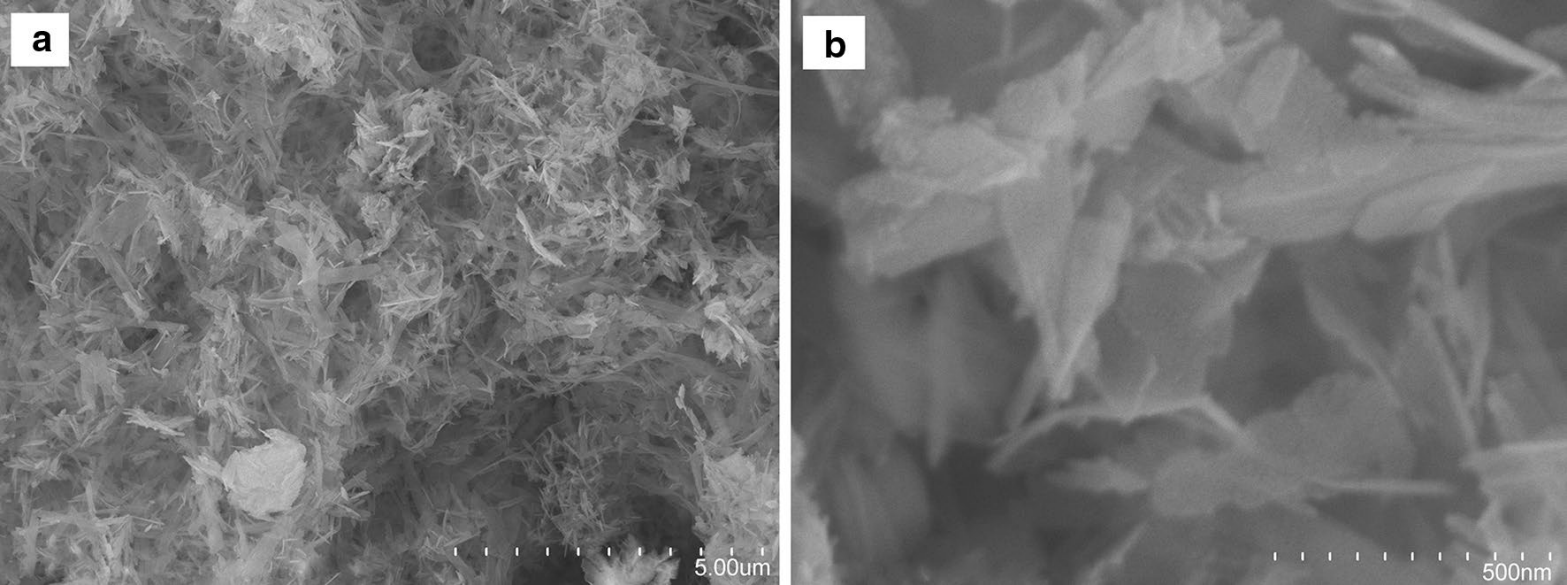
SEM images of HAP/GAC nanocomposite with different magnifications **a** 5.00 µm, **b** 500 nm

**Fig. 3:**
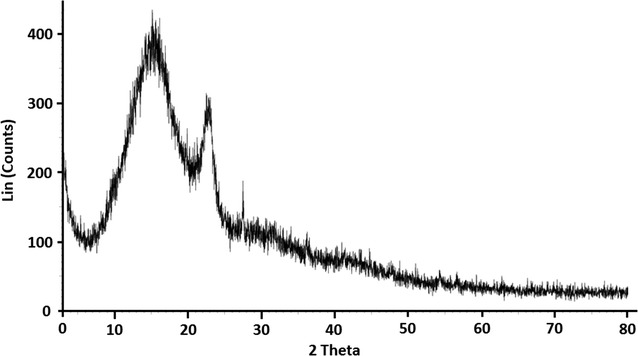
The XRD pattern of HAP/GAC

The SEM micrographs obtained for HAP/GAC nanocomposite with different magnifications are given in Fig. 2. The size and the morphology of the synthesized HAP nanoflakes on GAC was determined using scanning electron microscopy. The HAP comprised of high percentage of flake shape morphology and they are connected to a continuous mesh with a width less than 100 nm and varied in length. The SEM image obtained for HAP/GAC displays almost a uniform coating of nano HAP over GAC. Nevertheless, the coating of HAP nanoflakes have not blocked the pores of the GAC as previously observed [22], which can be considered as an advantage since most of the adsorption of GAC occurs at the pore sites in the GAC [47]. This composite was subjected to the X-ray diffractometry for further confirmation of the coating of HAP on GAC and given in Fig. 3.

The peaks observed with noise at the 2ϴ regions 26 and 42 can be assigned to GAC [48]. The peaks corresponding to neat HAP were not clear and this may be due to the low crystallinity of synthesized material. The observed XRD pattern for the HAP/GAC indicated that the HAP coated on the surface of GAC is not crystalline [26] but amorphous [22].

### Characterization of turmeric extract, HAP bi-coated granular activated carbon

**Fig. 4:**
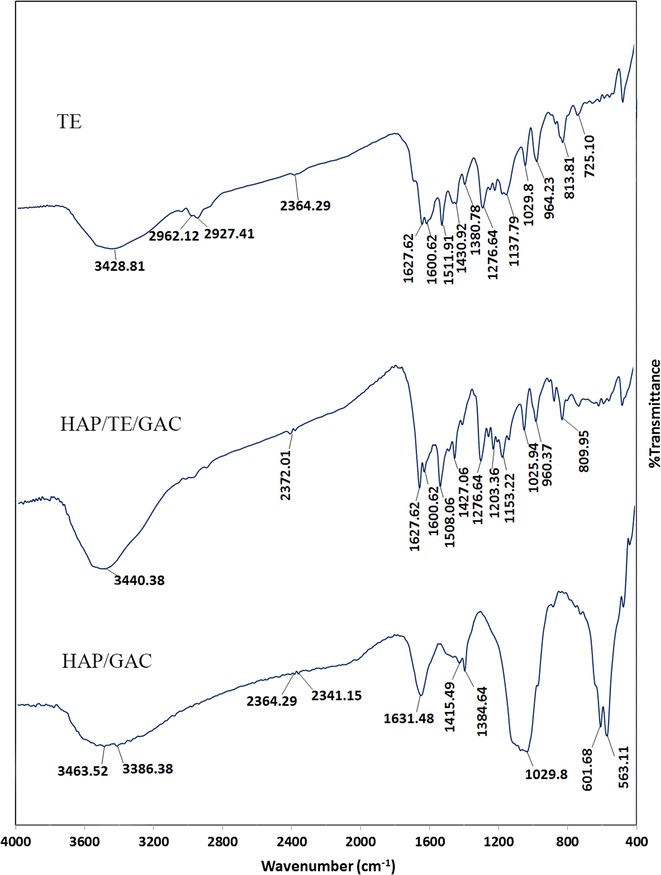
Comparison of FT-IR spectra of TE, HAP/GAC and HAP/TE/GAC

The HAP/GAC was further functionalized using the turmeric extract as given in the experimental section. The characterization of the curcuminoid pigments in the turmeric extract was carried out using FT-IR and UV–visible spectra. The comparison of FT-IR spectra for the original turmeric extract, HAP/TE/GAC and HAP/GAC are given in Fig. 4. The broad peak in the range of 3200–3600 cm^−1^ in neat turmeric extract can be attributed to the hydrogen bonded hydroxyl groups that are connected to the benzene rings in curcuminoids. The peak at 1627 cm^−1^ is due to the aryl substituted C=C bond. The peaks in the range of 2800–3000 cm^−1^ correspond to the C-H stretching of OCH_3_ groups [49]. The stretching vibrations of C=O group result the peak at 1600 cm^−1^ where it generally appear at much higher range [50]. The IR bands arising from curcuminoid pigments are having similar positions except for the region of 2500–3000 cm^−1^ as shown in Fig. 4.

Curcumin gives rise to a FT-IR band in the region of 2980–2850 cm^−1^ and demethoxycurcumin exhibits a band in the region of 2950–2850 cm^−1^ while bisdemethoxycurcumin does not show any characteristic band due to the absence of C–H stretching regions corresponding to the OCH_3_ groups [49]. Through the analysis of the FT-IR spectra in Fig. 4, it is clear that there are peaks in the region of 2850–2980 cm^−1^ in the turmeric extract justifying the presence of curcuminoids. The FT-IR spectrum for HAP/TE/GAC shows all peaks corresponding to the functional groups in the turmeric extract.

**Fig. 5:**
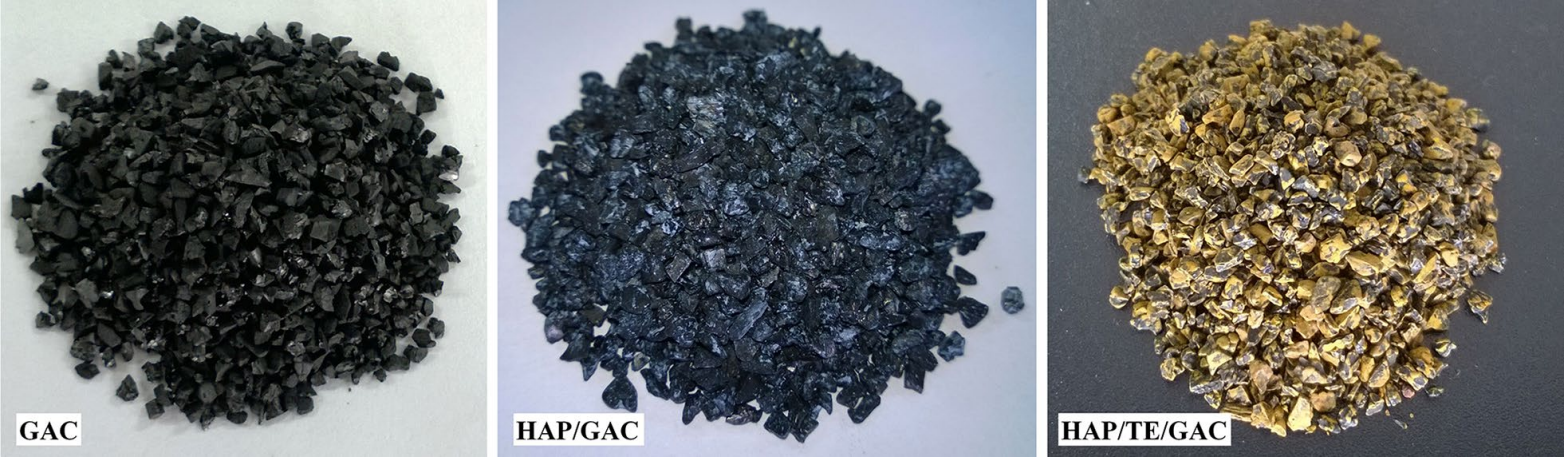
Comparison of physical appearance of GAC, HAP/GAC and HAP/TE/GAC

Figure 5 displays the physical appearance of the HAP/TE/GAC nanocomposite with the neat GAC and HAP/GAC. The neat GAC appears as black and this has become greyish with the coating of HAP. Successful coating of turmeric extract on HAP/GAC is clearly visible from the yellow colored HAP/TE/GAC composite.

**Fig. 6:**
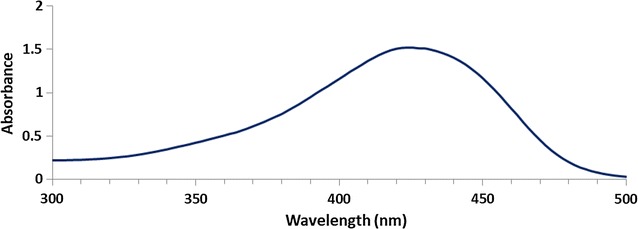
UV–visible spectrum of turmeric extract in ethanol

Turmeric extract was further characterized by UV–visible spectroscopy and the spectrum obtained for the turmeric extract in ethanol is shown in Fig. 6.

Curcuminoids dissolved in ethanol exhibits characteristic UV-visible peaks at 429 nm, 424 nm and 419 nm for curcumin, demethoxycurcumin and bisdemethoxycurcumin respectively [49]. As the sample contains all three pigments broader absorbance peak was observed between 418–432 nm with a maximum absorbance at 430 nm. Thus, it confirms the fact that turmeric extract used for the synthesis of nanocomposites contain curcuminoids.

**Fig. 7:**
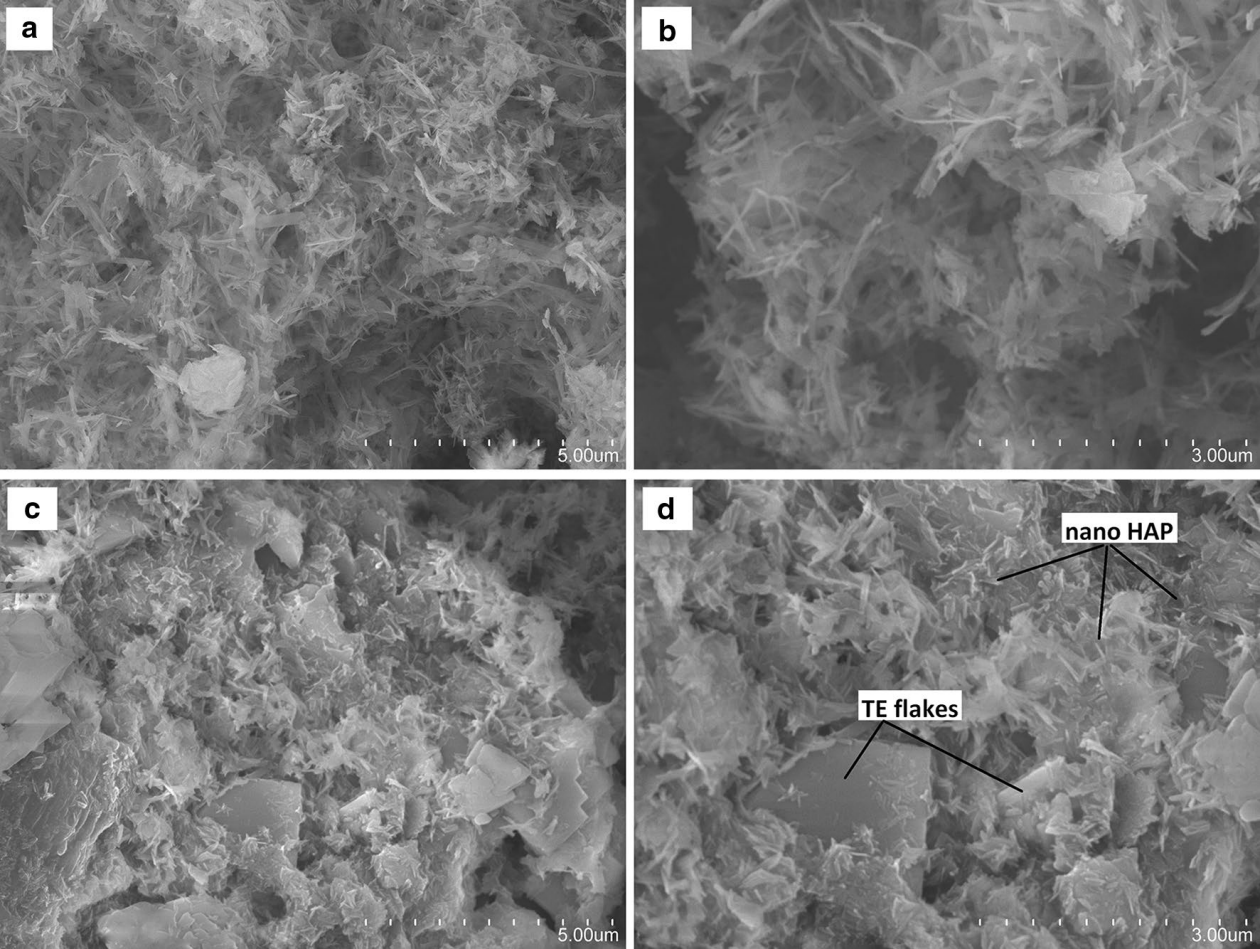
Comparison of SEM images of HAP/GAC **a** 5.00 µm, **b** 3.00 µm and HAP/TE/GAC **c** 5.00 µm, **d** 3.00 µm

The surface morphology of the HAP/TE/GAC was characterized using SEM (Fig. 7). Figure 7a and b represents only the HAP coated GAC and Fig. 7c and d represents turmeric extract coated HAP/GAC system (HAP/TE/GAC). Comparison of Fig. 7a–d indicates the presence of an additional solid material dispersed in between the HAP mesh. The deposition of turmeric extract had happened as flakes in between the mesh of nano HAP on the surface of the composite as shown in the diagram. The sizes of these flakes are almost in the micrometer range.

### Filtering of an *Escherichia coli* bacterial suspension using column technique

**Fig. 8:**
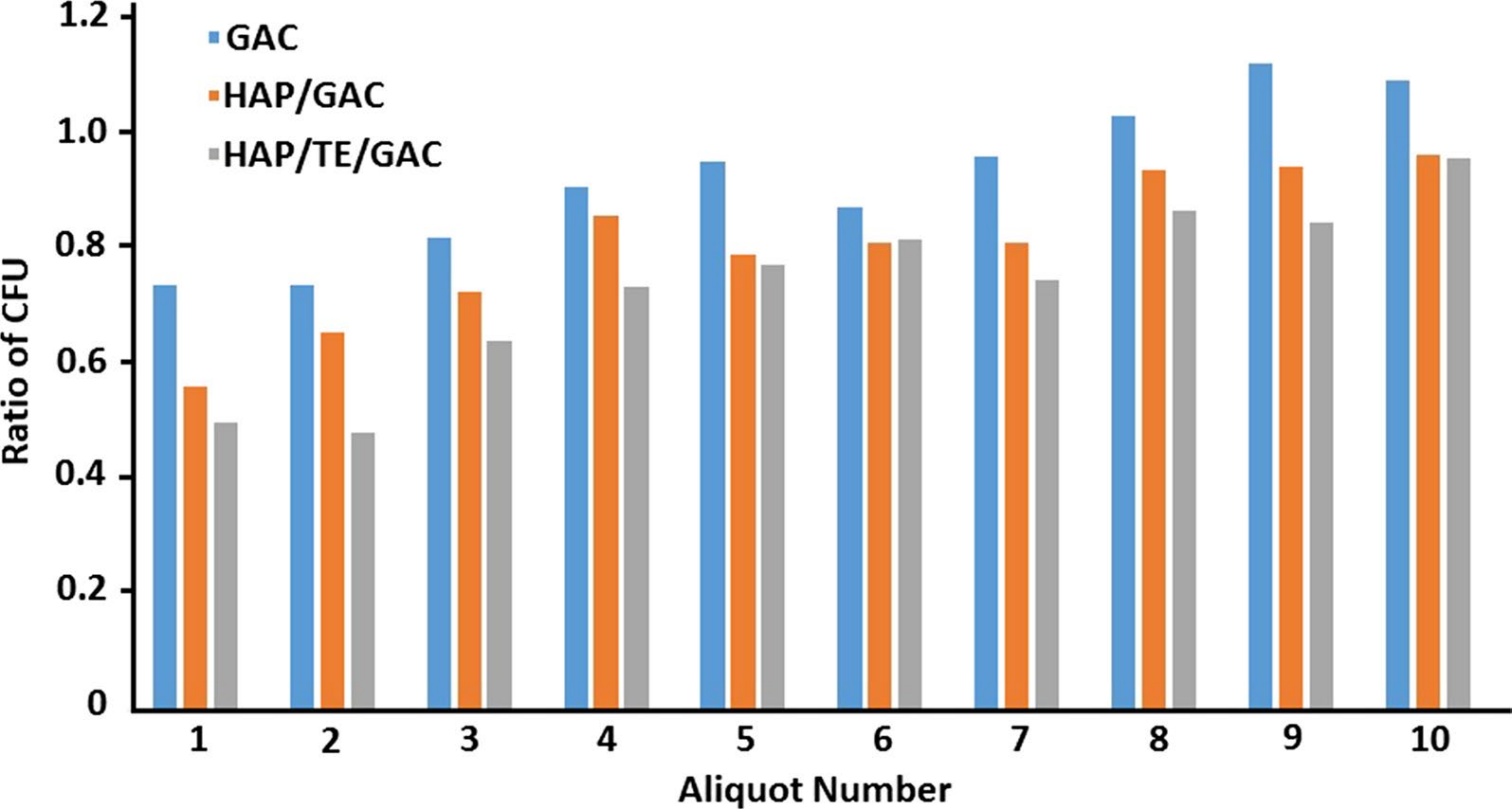
Variation of different ratios of CFUs with sequential aliquots of filtrate

The ability of removing bacteria by the composites was investigated using a known amount of *E. coli* bacterial suspension. This bacterial suspension was passed through columns packed with GAC, HAP/GAC and HAP/TE/GAC. The filtrate was collected as 10 mL aliquots as given in the methodology. Each fraction including original (initial) *E. coli* bacterial suspension was analyzed using impregnation method. The results obtained were the average number of colony forming units (CFUs) in 100 µL aliquot of the samples, by considering one bacterial cell as one CFU and the obtained results are summarized in Fig. 8.

$${\text{Ratio }}\;{\text{of}}\;{\text{CFUs}}\; = \; \frac{{{\text{Number }}\;{\text{of}}\;{\text{CFUs}}\;{\text{in }}\;{\text{the}}\;{\text{fraction}}\;{\text{of}}\;{\text{filtrate }}}}{{{\text{Number }}\;{\text{of}}\;{\text{CFUs }}\;{\text{in}}\;{\text{the }}\;{\text{initial }}\;{\text{bacterial }}\;{\text{suspension}}}}$$

According to the data given in Fig. 8, the lowest bacterial removing ability was exhibited by neat GAC. The ability of biofilm formation on GAC was also observed from results achieved during this experiment [26, 32]. Thus, it can be stated that GAC alone is not a good filter material for the filtration of bacterial contaminated water. Results also indicated a slight reduction of the antibacterial activity when going from first 10 mL to tenth 10 mL, which can be endorsed to the reduction of the active sites of nanomaterials and the accessibility of the antibacterial compounds for the binding of bacteria due to the filtering of a large volume of the bacterial suspension. According to results, the removing capacities shown by the nanocomposites of HAP/GAC and HAP/TE/GAC are slightly similar to each other. However, in almost in all the aliquots tested there was a small reduction of CFU in the composite HAP/TE/GAC. Similar antibacterial activity of the two filter materials, HAP/GAC and HAP/TE/GAC, is due to the presence of antibacterial material, HAP nanoflakes in both composites. Small increment in the antibacterial activity of the composite HAP/TE/GAC can be ascribed to the presence of curcuminoids. However, the absence of a significant change observed with CFU could be attributed to the fact that the amount of curcumin present in the turmeric extract used for the coating is less than the pure compound used in our previous study [26]. From this result, it is very clear that the synthesized nanocomposites can be categorized as bactericidal nanocomposites. The mechanism of action of curcumin in this system can be related to the inhibition of bacterial endotoxin induced cytokines secretion and related activation mechanisms, which directly suppresses bacterial cell growth as previously observed [39].

### Adsorption studies of Pb^2+^ ions

#### Effect of pH

**Fig. 9:**
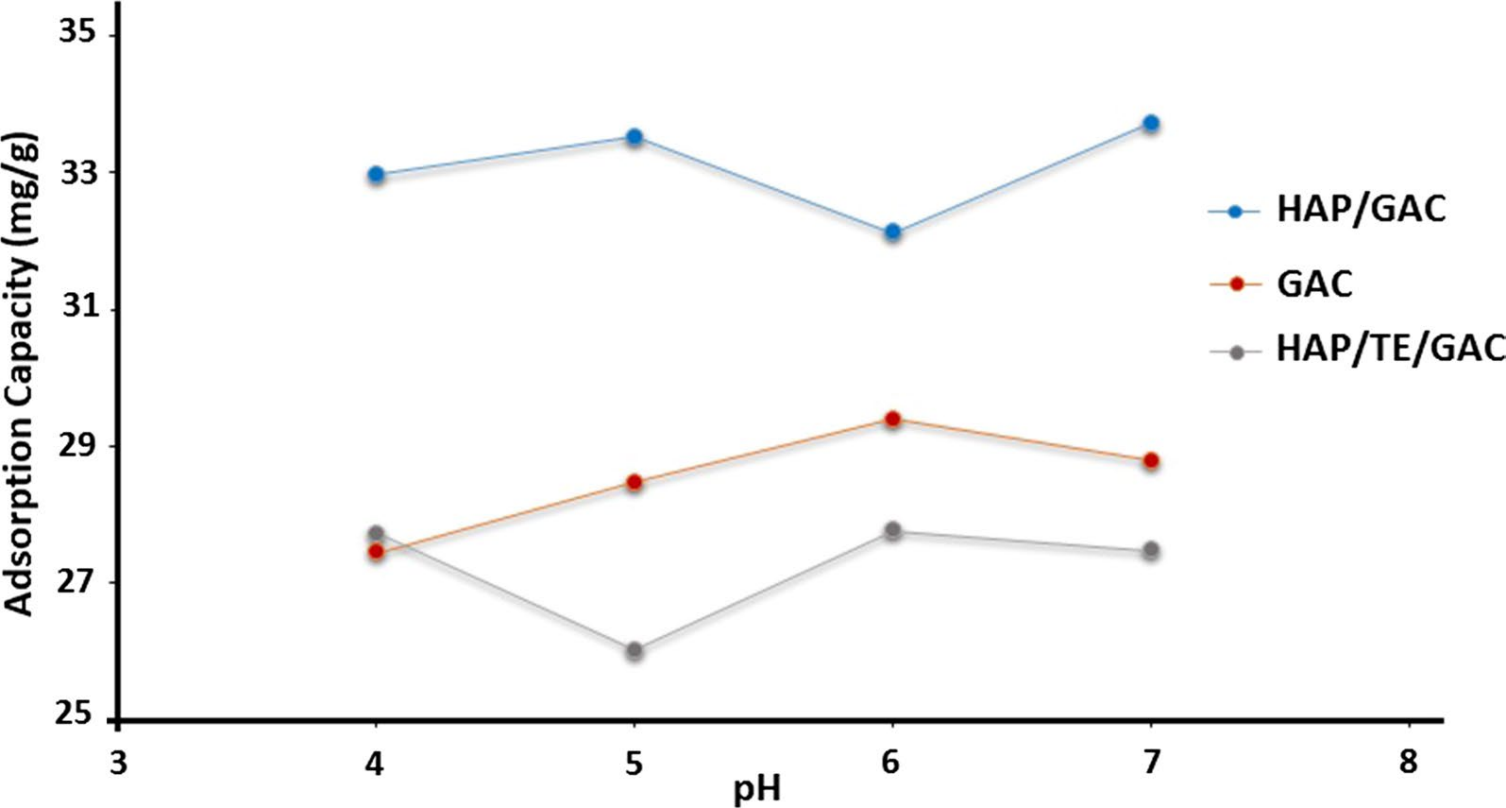
Comparison of the adsorption capacity of Pb^2+^ on GAC, HAP/GAC and HAP/TE/GAC with pH

The results of the pH dependence on adsorption studies for GAC, HAP/GAC and HAP/TE/GAC are given in Fig. 9. In this study, pH lower than 4 was not considered as HAP is not stable. According to the graph, the adsorption capacities of three composites show similar trends with pH. However, the highest adsorption was observed with HAP/GAC as observed in earlier studies. Slightly lower adsorption capacity of the composite HAP/TE/GAC compared to neat GAC can be explained having a thick coating of TE on the surface of the composite. In addition, it is also clear from the SEM (Fig. 7) that TE extract had deposited on the surface of HAP coated GAC as flakes blocking some pore areas reducing the exposure of the surface area present in the HAP/GAC composite. The adsorption studies were carried out at pH 6, as previously reported [51].

#### Effect of contact time

**Fig. 10:**
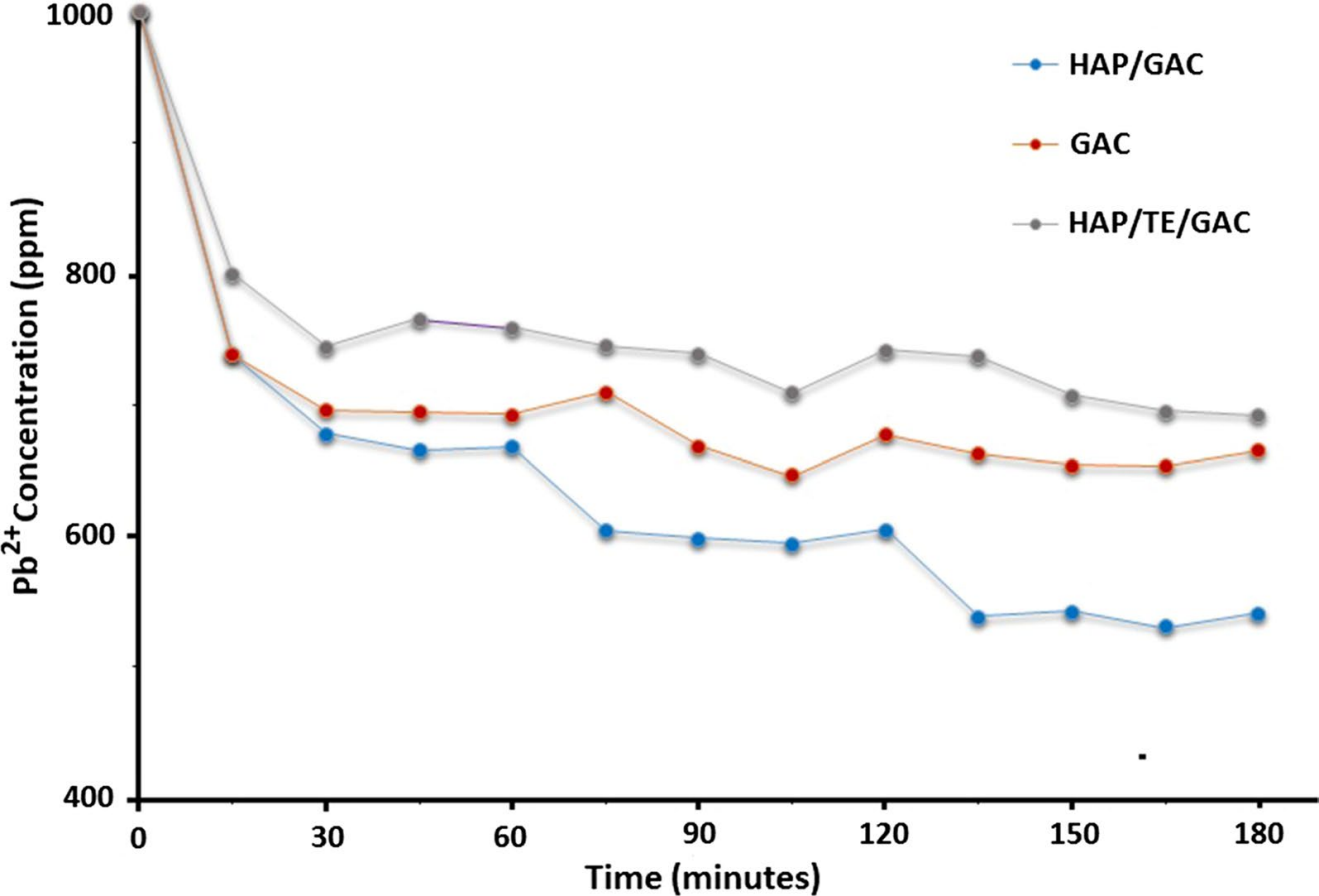
Comparison of Pb^2+^ ion concentration on GAC, HAP/GAC and HAP/TE/GAC with time

The adsorption studies were carried out to determine the effect of contact time using a 1000 ppm Pb^2+^ ion solution as given in the methodology. The results are shown in Fig. 10 and according to that, the equilibrium concentration was reached for both GAC and HAP/GAC after about 135 min whereas HAP/TE/GAC takes 150 min to reach the equilibrium. Thus, after 150 min all the three composites have reached the equilibrium concentration. Therefore 165 min was taken as the contact time for further adsorption studies [22].

#### Adsorption isotherms for neat GAC, HAP/GAC and HAP/TE/GAC composites

**Fig. 11:**
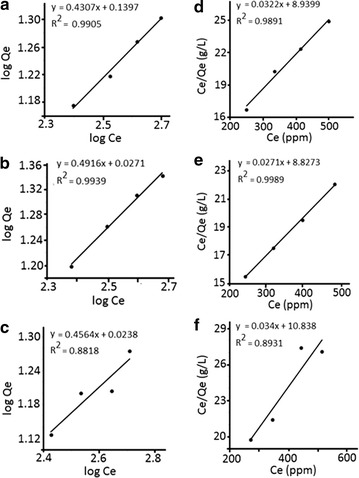
Freundlich isotherm of **a** neat GAC, **b** HAP/GAC, **c** HAP/TE/GAC and Langmuir isotherm of **d** neat GAC, **e** HAP/GAC, **f** HAP/TE/GAC

The adsorption isotherm studies of Pb^2+^ ions on neat GAC, HAP/GAC and HAP/TE/GAC were carried out by keeping the amount of composite constant at 1.0 g and by varying the Pb^2+^ ion concentrations from 400, 500, 600 and 700 ppm. In order to identify the best-suited model for the adsorption process, adsorption isotherms were plotted using both Freundlich and Langmuir models and results obtained are given in Fig. 11. According to adsorption isotherms, the equilibrium adsorption data correlates well with Freundlich isotherm model, which describes multilayer adsorption on heterogeneous surfaces, and Langmuir isotherm model that illustrates monolayer adsorption in the given Pb^2+^ ion concentration range.

According to the R^2^ values of the isotherm plots for GAC, the Langmuir isotherm and the Freundlich isotherm showed nearly the same correlation coefficient values greater than 0.98 at the given Pb^2+^ ion concentration. Thus, the adsorption process could be described well with both isotherm models as reported in previous related work [52]. The isotherm plots for HAP/GAC, Langmuir isotherm model (0.9989) and the Freundlich isotherm model (0.9939) gave almost the same correlation coefficient values in the given Pb^2+^ ion concentration. These values imply that the adsorption of Pb^2+^ on to HAP/GAC comply with both monolayer and multilayer adsorption on heterogeneous surface [22, 40, 52]. However, correlation coefficient values obtained for HAP/TE/GAC composite showed slightly lower R^2^ for both Langmuir and Freundlich models compared with neat GAC and HAP/GAC composite. This may be assumed to the presence of three types of adsorbate sites with different affinities towards Pb^2+^ ions.

The adsorption constants were calculated using the slope and the intercept of the linear trendlines for the adsorption of Pb^2+^ by GAC, HAP/GAC and HAP/TE/GAC composites, are given in Table 1. The detailed adsorption data are included in the supporting materials. The adsorption capacity of HAP/TE/GAC was 29.4 mg of Pb^2+^ ions per 1 g of the composite. The results show that though HAP/GAC has the highest adsorption capacity among the composites, HAP/TE/GAC can also be used effectively in removing Pb^2+^ ions from aqueous solutions. In addition this composite is capable of removing bacteria and hence offsetting any reduced effect in metal ion removal and proving to be a multifunctional nanocomposite to purify water.

**Table 1:** Langmuir and Freundlich adsorption isotherm constants for the adsorption of Pb^2+^ ions on to GAC, HAP/GAC and HAP/TE/GAC

Material	Langmuir adsorption isotherm constants			Freundlich adsorption isotherm constants		
	q_m_ (mg/g)	K (L/mg)	R^2^	K_f_	n	R^2^
HAP/GAC	36.9 (± 0.8)	3.1 × 10^−3^ (± 1.1 × 10^−4^)	0.9989	1.1 (± 0.2)	2.0 (± 0.1)	0.9939
GAC	31.1 (± 2.3)	3.6 × 10^−3^ (± 4.6 × 10^−4^)	0.9891	1.4 (± 0.2)	2.3 (± 0.2)	0.9905
HAP/TE/GAC	29.4 (± 7.2)	3.1 × 10^−3^ (± 1.2 × 10^−3^)	0.8931	1.1 (± 0.7)	2.2 (± 0.6)	0.8818

## Conclusion

From the adsorption data it can be stated that 100 g of HAP/TE/GAC is capable of removing 2941.2 mg of Pb^2+^ ions from aqueous solution. It is also evident that the synthesized nanocomposite can be categorized as a bactericidal nanocomposite. Thus HAP/TE/GAC composite is capable of maintaining inherent properties of GAC while enhancing its capacity to remove heavy metals and bacterial contaminants. The biocompatible naturally occuring turmeric extract and HAP based activated carbon nanocomposite can be a strong contender to replace traditional silver and nanosilver based antibacterial filters which are detrimental to the environment due to various reasons.

## Additional file

**Additional file 1.** Additional tables.
