# Supplementary material for: Synthesis of multifunctional activated carbon nanocomposite comprising biocompatible flake nano hydroxyapatite and natural turmeric extract for the removal of bacteria and lead ions from aqueous solution
Source: Chem Cent J. 2018 Feb 21;12:18. doi: 10.1186/s13065-018-0384-7 (PMC5821621; doi:10.1186/s13065-018-0384-7)
Supplement: Supplementary file 1 — Additional file 1. Additional tables. [file 13065_2018_384_MOESM1_ESM.docx]

# Additional file

# Effect of pH on adsorption studies for lead ions (Pb^2+^) by neat GAC, HAP coated GAC (HAP/GAC) and HAP turmeric extract bi-coated GAC (HAP/TE/GAC)

## Lead (Pb^2+^) adsorption capacity of GAC with pH

| **pH** | **Initial concentration (C_0_) (ppm)** | **Equilibrium concentration (C_e_) (ppm)** | | | | **Adsorption capacity (mg/g)** |
| --- | --- | --- | --- | --- | --- | --- |
|  |  | **Trial 1** | **Trial 2** | **Trial 3** | **Average** |  |
| 4.0 | 1000 | 712 | 736.1 | 728.3 | 725.467 | 27.453 |
| 5.0 | 1000 | 700.3 | 690.4 | 755 | 715.233 | 28.477 |
| 6.0 | 1000 | 689.3 | 690.9 | 737.5 | 705.9 | 29.41 |
| 7.0 | 1000 | 715.3 | 714.3 | 706.5 | 712.033 | 28.797 |

## Lead (Pb^2+^) adsorption capacity of HAP/GAC with pH

| **pH** | **Initial concentration (C_0_) (ppm)** | **Equilibrium concentration (C_e_) (ppm)** | | | | **Adsorption capacity (mg/g)** |
| --- | --- | --- | --- | --- | --- | --- |
|  |  | **Trial 1** | **Trial 2** | **Trial 3** | **Average** |  |
| 4.0 | 1000 | 675.5 | 684 | 651.3 | 670.267 | 32.973 |
| 5.0 | 1000 | 673 | 658.9 | 662.1 | 664.667 | 33.533 |
| 6.0 | 1000 | 662.2 | 693 | 680.5 | 678.567 | 32.143 |
| 7.0 | 1000 | 678.7 | 647 | 662 | 662.567 | 33.743 |

## Lead (Pb^2+^) adsorption capacity of HAP/TE/GAC with pH

| **pH** | **Initial concentration (C_0_) (ppm)** | **Equilibrium concentration (C_e_) (ppm)** | | | | **Adsorption capacity (mg/g)** |
| --- | --- | --- | --- | --- | --- | --- |
|  |  | **Trial 1** | **Trial 2** | **Trial 3** | **Average** |  |
| 4.0 | 1000 | 717.1 | 737.2 | 714.2 | 722.833 | 27.717 |
| 5.0 | 1000 | 722.6 | 741.2 | 755.6 | 739.8 | 26.02 |
| 6.0 | 1000 | 755.2 | 710.1 | 701.6 | 722.3 | 27.77 |
| 7.0 | 1000 | 735.6 | 726.4 | 713.8 | 725.267 | 27.473 |

# Data for the plot of determination of contact time of GAC, HAP/GAC, and HAP/TE/GAC

| **Time (mins)** | **Concentration with time (ppm)** | | |
| --- | --- | --- | --- |
|  | **GAC** | **HAP/GAC** | **HAP/TE/GAC** |
| 0 | 1000 | 1000 | 1000 |
| 15 | 738.033 | 738.2 | 799.5 |
| 30 | 695.167 | 677.767 | 743.867 |
| 45 | 694.333 | 665.167 | 765.267 |
| 60 | 692.033 | 667.267 | 758.4 |
| 75 | 709.133 | 603.467 | 744.433 |
| 90 | 668.1 | 597.9 | 738.767 |
| 105 | 646.233 | 593.933 | 708.367 |
| 120 | 676.967 | 604.2 | 741.433 |
| 135 | 662.2 | 537.667 | 736.467 |
| 150 | 653.667 | 542.167 | 706.933 |
| 165 | 652.833 | 530.333 | 694.867 |
| 180 | 665.067 | 540.867 | 692.3 |

# Data for Adsorption Isotherms

## Data for Freundlich adsorption isotherm for adsorption of Pb^2+^ ions to GAC

| **Initial concentration (C_o_) (ppm)** | **Equilibrium concentration (C_e_) (ppm)** | **(C_o_-C_e_) (ppm)** | **Mass of the adsorbate (mg)** | **(Q_e_) (mg/g)** | **log C_e_** | **log Q_e_** |
| --- | --- | --- | --- | --- | --- | --- |
| 400 | 249.867 | 150.133 | 15.013 | 15.013 | 2.398 | 1.176 |
| 500 | 334.4 | 165.6 | 16.56 | 16.56 | 2.524 | 1.219 |
| 600 | 413.95 | 186.05 | 18.605 | 18.605 | 2.617 | 1.27 |
| 700 | 498.92 | 201.08 | 20.108 | 20.108 | 2.698 | 1.303 |

## Data for Langmuir adsorption isotherm for adsorption of Pb^2+^ ions to GAC

| **Initial concentration (C_o_) (ppm)** | **Equilibrium concentration (C_e_) (ppm)** | **(C_o_-C_e_) (ppm)** | **Mass of the adsorbate (mg)** | **(Q_e_) (mg/g)** | **C_e_/Q_e_ (g/dm^3^)** |
| --- | --- | --- | --- | --- | --- |
| 400 | 249.867 | 150.133 | 15.013 | 15.013 | 16.643 |
| 500 | 334.4 | 165.6 | 16.56 | 16.56 | 20.193 |
| 600 | 413.95 | 186.05 | 18.605 | 18.605 | 22.249 |
| 700 | 498.92 | 201.08 | 20.108 | 20.108 | 24.812 |

## Data for Freundlich adsorption isotherm for adsorption of Pb^2+^ ions to HAP/GAC

| **Initial concentration (C_o_) (ppm)** | **Equilibrium concentration (C_e_) (ppm)** | **(C_o_-C_e_) (ppm)** | **Mass of the adsorbate (mg)** | **(Q_e_) (mg/g)** | **log C_e_** | **log Q_e_** |
| --- | --- | --- | --- | --- | --- | --- |
| 400 | 242.867 | 157.133 | 15.713 | 15.713 | 2.385 | 1.196 |
| 500 | 317.867 | 182.133 | 18.213 | 18.213 | 2.502 | 1.260 |
| 600 | 396.183 | 203.817 | 20.382 | 20.382 | 2.598 | 1.309 |
| 700 | 480.917 | 219.083 | 21.908 | 21.908 | 2.682 | 1.341 |

## Data for Langmuir adsorption isotherm for adsorption of Pb^2+^ ions to HAP/GAC

| **Initial concentration (C_o_) (ppm)** | **Equilibrium concentration (C_e_) (ppm)** | **(C_o_-C_e_) (ppm)** | **Mass of the adsorbate (mg)** | **(Q_e_) (mg/g)** | **C_e_/Q_e_ (g/dm^3^)** |
| --- | --- | --- | --- | --- | --- |
| 400 | 242.867 | 157.133 | 15.713 | 15.713 | 15.456 |
| 500 | 317.867 | 182.133 | 18.213 | 18.213 | 17.452 |
| 600 | 396.183 | 203.817 | 20.382 | 20.382 | 19.438 |
| 700 | 480.917 | 219.083 | 21.908 | 21.908 | 21.951 |

## Data for Freundlich adsorption isotherm for adsorption of Pb^2+^ ions to HAP/TE/GAC

| **Initial concentration (C_o_) (ppm)** | **Equilibrium concentration (C_e_) (ppm)** | **(C_o_-C_e_) (ppm)** | **Mass of the adsorbate (mg)** | **(Q_e_) (mg/g)** | **log C_e_** | **log Q_e_** |
| --- | --- | --- | --- | --- | --- | --- |
| 400 | 266.45 | 133.55 | 13.355 | 13.355 | 2.426 | 1.126 |
| 500 | 341.85 | 158.15 | 15.815 | 15.815 | 2.534 | 1.199 |
| 600 | 440.283 | 159.717 | 15.972 | 15.972 | 2.644 | 1.203 |
| 700 | 512 | 188 | 18.8 | 18.8 | 2.709 | 1.274 |

## Data for Langmuir adsorption isotherm for adsorption of Pb^2+^ ions to HAP/TE/GAC

| **Initial concentration (C_o_) (ppm)** | **Equilibrium concentration (C_e_) (ppm)** | **(C_o_-C_e_) (ppm)** | **Mass of the adsorbate (mg)** | **(Q_e_) (mg/g)** | **C_e_/Q_e_ (g/dm^3^)** |
| --- | --- | --- | --- | --- | --- |
| 400 | 266.45 | 133.55 | 13.355 | 13.355 | 19.951 |
| 500 | 341.85 | 158.15 | 15.815 | 15.815 | 21.616 |
| 600 | 440.283 | 159.717 | 15.972 | 15.972 | 27.567 |
| 700 | 512 | 188 | 18.8 | 18.8 | 27.234 |

# Data for *Escherichia coli* bacteria filtering through GAC, HAP/GAC and HAP/TE/GAC

| **Fraction of filtrate** | **Number of colony forming units (CFU) in 100 µL** | | |
| --- | --- | --- | --- |
|  | **GAC** | **HAP/GAC** | **HAP/TE/GAC** |
| Initial solution | 1055 | 1055 | 1055 |
| 1^st^ 10 mL | 772 | 590 | 526 |
| 2^nd^ 10 mL | 773 | 687 | 506 |
| 3^rd^ 10 mL | 858 | 761 | 673 |
| 4^th^ 10 mL | 953 | 898 | 770 |
| 5^th^ 10 mL | 997 | 830 | 810 |
| 6^th^ 10 mL | 916 | 850 | 857 |
| 7^th^ 10 mL | 1008 | 850 | 782 |
| 8^th^ 10 mL | 1082 | 981 | 910 |
| 9^th^ 10 mL | 1175 | 990 | 886 |
| 10^th^ 10 mL | 1146 | 1010 | 1005 |
